# Enabling Secure XMPP Communications in Federated IoT Clouds Through XEP 0027 and SAML/SASL SSO

**DOI:** 10.3390/s17020301

**Published:** 2017-02-07

**Authors:** Antonio Celesti, Maria Fazio, Massimo Villari

**Affiliations:** 1 Scientific Research Organisational Unit, University of Messina, 98122 Messina, Iatly; 2 Department of Engineering, University of Messina, 98166 Messina, Italy; mfazio@unime.it (M.F.); mvillari@unime.it or massimo.villari@irccsme.it (M.V.); 3 IRCCS Centro Neurolesi Bonino Pulejo, 98124 Messina, Italy

**Keywords:** IoT, Cloud computing, federation, security, XMPP

## Abstract

Nowadays, in the panorama of Internet of Things (IoT), finding a right compromise between interactivity and security is not trivial at all. Currently, most of pervasive communication technologies are designed to work locally. As a consequence, the development of large-scale Internet services and applications is not so easy for IoT Cloud providers. The main issue is that both IoT architectures and services have started as simple but they are becoming more and more complex. Consequently, the web service technology is often inappropriate. Recently, many operators in both academia and industry fields are considering the possibility to adopt the eXtensible Messaging and Presence Protocol (XMPP) for the implementation of IoT Cloud communication systems. In fact, XMPP offers many advantages in term of real-time capabilities, efficient data distribution, service discovery and inter-domain communication compared to other technologies. Nevertheless, the protocol lacks of native security, data confidentiality and trustworthy federation features. In this paper, considering an XMPP-based IoT Cloud architectural model, we discuss how can be possible to enforce message signing/encryption and Single-Sign On (SSO) authentication respectively for secure inter-module and inter-domain communications in a federated environment. Experiments prove that security mechanisms introduce an acceptable overhead, considering the obvious advantages achieved in terms of data trustiness and privacy.

## 1. Introduction

Nowadays, the combination of Cloud computing and Internet of Things (IoT) technologies is pursuing new opportunities in delivering services, representing a strategic approach for IT operators to increase their business. The emerging business perspectives coming from IoT are pushing private, public, and hybrid Cloud providers to integrate their systems with embedded IoT devices (including sensors and actuators) in order to provide along with the traditional Infrastructure, Platform, and Software as a Services (IaaS, PaaS, SaaS) even a new type of transversal service level, that is defined *IoT as a Service* (IoTaaS). Indeed, IoTaaS can be considered as a service model to provide IoT resources in terms of Infrastructure, Platform, and Software. As a consequence, new types of providers that combine Cloud computing solutions with IoT are rising. The term IoT Cloud is considered to indicate a new type of distributed system consisting of a set of IoT devices interconnected with a remote Cloud infrastructure, platform, or software through the Internet able to provide IoTaaS. In this context, the concept of IoT Cloud federation is becoming more and more popular. It is defined as a mesh of IoT Cloud providers that are interconnected to provide a universal decentralized sensing and actuating environment where everything is driven by constraints and agreements in an ubiquitous infrastructure [1]. In such an emerging scenario, IoT Cloud providers require to carry out secure inter-module and inter-domain communications over the Internet.

Until now, well-known communication technologies adopted in pervasive systems such the Constrained Application Protocol (CoAP) [2] and AllJoyn [3] are not adequate to meet the requirements IoT Clouds deployed over large-scale Internet scenarios because they are conceived for Local Area Network (LAN) environments. Considering IoT Clouds spread over the Internet, the trend has been to base their communication systems on well-known client-server web service technologies such as the Representational State Transfer (REST) and the Simple Object Access Protocol (SOAP). This model has succeeded until now, however, due to the increasingly degree of complexity and interactivity that IoT Cloud architectures need to address, the achievement of both interactivity and security capabilities is not trivial at all. In fact, both REST and SOAP technologies present the following disadvantages: (i) they are based on request/response patterns; (ii) they do not provide any native asynchronous interaction; (iii) their polling does not scale well and it is not real-time; (iv) they require a two-way data exchange. Consequently, web services make complicated (i) the presence (availability) and discovery of software modules and services; (ii) many-to-many distribution patterns; (iii) asynchronous and multi-step calls to remote services; (iv) federation with third-party providers and services especially behind firewalls. In such a context the Open Interconnect Consortium (OIC) specification 1.1 [4] of the Open Connectivity Foundation (OCF) bases the communications between devices on COaP and REST technologies with obvious limitations in message management and interactivity.

For these reasons, most operators are looking at alternative communication protocols including the Message Queue Telemetry Transport (MQTT) [5], Advanced Message Queuing Protocol (AMQP) [6], Data Distribution Service (DDS) [7] and Extensible Messaging and Presence Protocol (XMPP) [8]. As recently discussed in [9], MQTT seams to be the best solution in terms of latency. However, things are different considering both message management and security. In fact, MQTT is a client/server public/subscribe technology that does not support direct end-to-end and multicast messaging. As far as security, MQTT only supports the Simple Authentication and Security Layer (SASL) [10] and Transport Layer Security (TLS) mechanisms respectively for the authentication and for the encryption of the communication channel. OneM2M (i.e., a global organization that creates requirements, architecture, API specifications, security solutions and interoperability for Machine-to-Machine and IoT technologies) only supports client/server communications through Hypertext Transfer Protocol (HTTP), COaP and MQTT bindings.

On the contrary, XMPP (Request for Comments (RFC) 6120 [11]) seams to be more suitable for addressing the requirements of emerging IoT Cloud providers in terms of both message management and security. XMPP is an open-standard communications protocol for message-oriented middleware based on the XML (Extensible Markup Language). On one hand the XMPP is able to overcome the disadvantages of MQTT in terms of management because, apart from the public/subscribe communication, it also supports end-to-end and multicast communications. On the other hand, considering security and privacy, similarly to MQTT, XMPP supports only SASL and TLS capabilities, but it lacks of native advanced security features for addressing the security requirements of emerging federation-enabled IoT Cloud scenarios. Such requirements include:

- End-to-End and multicast message signing/encryption for inter-module communication;
- Cloud-to-Cloud authentication for inter-domain authentication.

In this paper, it is discussed how XMPP can be adopted for the development of a secure communication system addressing the aforementioned requirements. In particular, starting from a generic message-oriented architectural model, the main security issues regarding the adoption of XMPP in federation-enabled IoT Clouds are analysed and a discussion is provided regarding how they can be mitigated through the integration of both Security Assertion Markup Language (SAML)/SASL Single-Sign On (SSO) authentication and XMPP Extension Protocol (XEP) 0027 end-to-end and multicast message signing/encryption extensions respectively to carry out inter-module and inter-domain communications. Experiments conducted in a real testbed, at the healthcare clinical and research centre IRCCS - Centro Neurolesi “Bonino Pulejo” (Messina, Italy), prove the the overhead introduced by the aforementioned XMPP security extensions are acceptable in terms of transmission response time.

The rest of the paper is organized as follows. Section 2 describes related work. Section 3 describes an architectural model that it is used as a reference in order to discuss an XMPP-based communication system for IoT Clouds. Section 4 presents the advantages in using XMPP-based communication systems for complex federation-enabled IoT Cloud architectures. In Section 5, the limits of XMPP in terms of security are highlighted. In particular, XMPP security integrations enabling secure inter-module and inter-domain communications in federated IoT Cloud environments are discussed in Section 6. Experiments on a real testbed are discussed in Section 7. Conclusion and lights to the future are discussed in Section 8.

## 2. Related Work

Nowadays, Cloud computing is a widely debated topic. In fact, there are many scientific works that apply the Cloud technology to different fields including energy efficiency [12,13], service provisioning [14], storage [15], IoT [1] and so on. In particular, several works have been recently proposed regarding IoT Cloud architectures [16,17,18]. In this context, security in IoT data transmission is a current issue. An empirical study on security in future IoT service environments is proposed in [19]. In particular, the authors conducted a study on the distribution of information security roles. A generic authentication approach for RESTful IoT protocols which consider scalability and resource-restrictiveness constraints stemming from the architectural style of REST and IoT environments is proposed in [20]. An identity and access control requirement analysis for IoT is discussed in [21]. A dynamic hierarchical role-based access control model, useful in Cloud services aimed at mobile Internet is described in [22]. In particular, the authors introduce an interesting security model with a self-adaptive schema that enables a system to automatically meet the environmental parameters, hence offering the corresponding protections. A study on secure distributed detection problems under energy constraint for IoT-oriented sensor networks is proposed in [23]. In particular, authors focus on how to optimize the key thresholds for estimating the channel gain in Channel-Aware Encryption (CAE). In [24], authors methodically assess the possible impacts of a specific class of Amplified Reflection Distributed Denial of Service (AR-DDoS) attacks against IoT. In [25] authors propose a protocol designed for multi-hop communications between Device-to-Device (D2D)-enabled terminals equipped with light-weight security mechanisms. It is meant to address the communication requirements of User Equipments (UEs) inside the mobile network coverage, and those of UEs that suffer from scarce radio coverage. In terms of performance, they analyse connectivity and security in the multi-hop D2D network, taking into account the interference created by the transmission of beacon signals during the discovery phase of a wide network. In [26], authors present a study on the importance of the secrecy outage performance of wireless communications under eavesdropper collusion where the physical layer security is adopted to counteract attacks. Based on the classical probability theory, authors first conduct an analysis on the secrecy outage of a simple non-colluding case in which eavesdroppers do not independently collude and operate. In [27], the authors propose a novel proxy-based authentication and key establishment protocol, which is lightweight and suitable to safeguard sensitive data generated by resource-constrained devices in IoT-enabled Ambient Assisted Living (AAL) systems. A security mechanism that deals with the requirements of authentication, integrity, confidentiality, non-repudiation, and access control in XMPP sensor networks is proposed in [28]. International Standardization Organization (ISO), International Electrotechnical Commission (IEC), Institute of Electrical and Electronics Engineers (IEEE) 21451 messages are exchanged based on the public/subscribe model using an extended security simple object access protocol over XMPP. In [29], the authors present the VIRTUS middleware, a piece of IoT middleware relying on the open XMPP protocol to provide secure event-driven communications within an IoT scenario. Leveraging the standard security features provided by XMPP, the piece of middleware offers a reliable and secure communication channel for distributed applications, protected with both authentication (through TLS) and encryption (through SASL) mechanisms. The proposed architecture provides the possibility to isolate an instance of VIRTUS, allowing the exchange of data only within a private network. Differently from the aforementioned scientific works, this paper specifically focuses on the security issues regarding the communication system of a federation-enabled IoT Cloud environment.

## 3. An Architectural Model for Federated IoT Cloud Environments

In order to describe the advantages of an XMPP communication system in an IoT environment, the Message Oriented Middleware for Cloud Computing (MOM4C) architectural model [30] is considered. MOM4C is designed according to the message-oriented paradigm, in order to provide an efficient communication system among different distributed components. MOM4C allows a highly cohesive, decoupled system deployment. It also decouples the performance of subsystems. In fact, they can be independently scaled, with little or no disruption of performance into the other subsystems. With reference to the management of unpredictable activity overloads in a subsystem, the message-oriented model allows to accept a message when it is ready, rather than being forced to accept it. Moreover, MOM4C adds several important features, that are strategic for the business of IoT Clouds. Its major benefits includes:

- **Modularity**: The middleware can be quickly extended in terms of available utilities and it can be easily customized in order to suits a specific IoT Cloud scenario.
- **Polymorphism**: Each distributed entity in the system can play different roles according to the system requirements.
- **Security**: An indispensable requirement for large-scale IoT Clouds is security, especially in business scenarios. Security has to be natively addressed at any level of communication (intra-module, inter-module, and inter-domain), providing guarantees in terms of data confidentiality and data integrity
- **Federation**: It is a strategic approach to promote collaboration among cooperating IoT Cloud providers.

**Figure 1:**
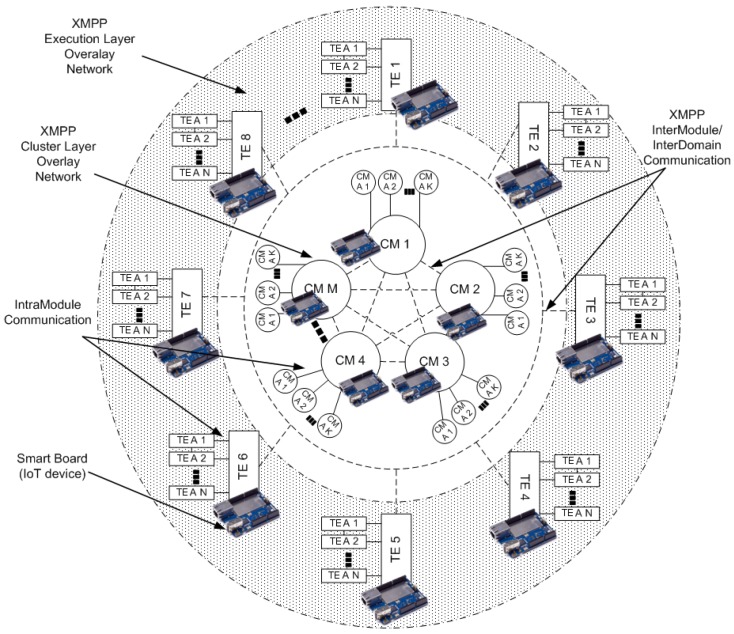
MOM4C scheme adapted for a an IoT scenario.

As depicted in Figure 1, MOM4C is based on a distributed architecture, organized in two layers, that are the Cluster Layer (CL) and the Execution Layer (EL). The Cluster Layer represent the “core” of MOM4C. It consists of an overlay network of decentralized Cluster Manager (CM) nodes. Each CM, by means of software agents (CM A), is responsible for the working activities of Task Executor (TE) nodes belonging to the cluster. The EL is composed of TEs, which are intended to perform operative tasks. It means that they do not instantiate all the services and utilities available in MOM4C, but they download code, initialize and configure services, launch software agents (TE A) whenever they receives instructions from the CM. According to the specific code in execution at TEs, we different characterizations of the EL are possible. Another important feature of MOM4C is the polymorphic nature of nodes. At different times, each IoT device can serve as CM or TE. However, only an IoT device in a cluster is elected as CM and actively works for managing the whole cluster. Some other IoT devices are elected as “passive CMs”, which are redundant CMs that can quickly replace the active CM if it fails. This approach improves the fault tolerance of the CL. The size of the cluster depends on the system workload and it can dynamically change according to the specific elasticity requirements of the system.

**Figure 2:**
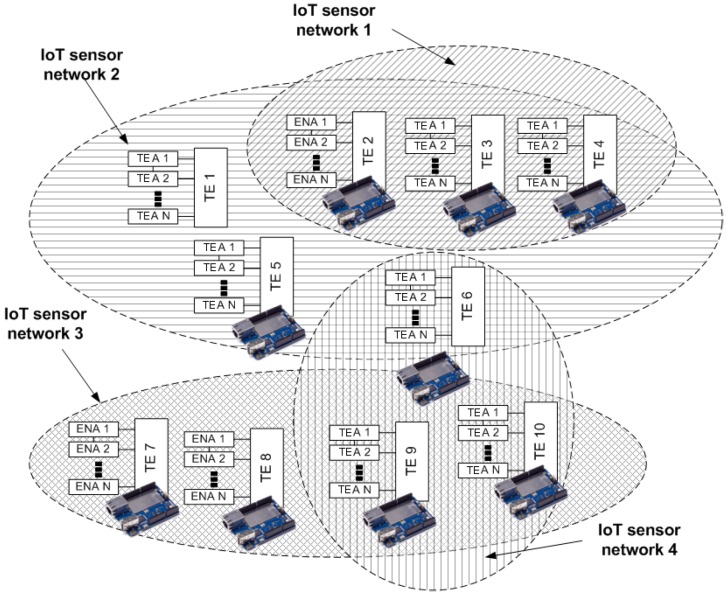
Hybrid executor node layer composition in an IoT scenario.

Considering an IoT scenario, CM nodes can be deployed in IoT devices such as Raskberry, Arduino, and so on. Whereas an EL represents an IoT sensor network. Thus MOM4C enables several simultaneous overlay sensor networks. TE nodes can be customized by means of the installation of external software agents exploiting the container technology that enables IoT devices to exploit a lightweight mechanism of virtualization [31]. TE nodes can belong to one or more ELs, i.e., they can belong to different IoT sensor networks. Such a concept is better explained in Figure 2. TE 2, 3, and 4 are IoT devices belonging to IoT sensor network 2. At the same time, TE 1, 2, 3, 4, 5, 6 belong to IoT sensor network 2. TE 7, 8, 9, and 10 belong to IoT sensor network 3. Finally, TE 6, 9, and 10 belong to IoT sensor network 4.

MOM4C supports three types of communications:

- **IntraModule Communication**: It allows information exchange inside each node of the architecture.
- **InterModule Communication**: It governs communications between CMs and TEs and vice-versa.
- **InterDomain Communication**: It enables the communication between CMs belonging to different administrative domains, hence enabling IoT federation scenarios.

In order to ensure as much as possible middleware modularity, the tasks running on each node are mapped on different processes which communicate each other by means of an Inter Process Communication (IPC) or InterModule communication. According to the message-oriented design of MOM4C, InterModule and InterDomain communications are based on an Instant Messaging and Presence (IMP) protocol. A presence system allows participants to subscribe each other and to be notified about changes in their state. On the other hand, instant messaging enables the exchange of messages between a set of participants in near real time. InterDomain communications among different administrative domains are managed considering federation agreements. Federation allows IoT Cloud providers to “lend” and “borrow” IoT devices. Thus, a CM of a domain is able to control one or more TEs belonging to other federated domains.

## 4. Why Does XMPP Suit IoT?

Considering the MOM4C architectural model, this Section specifically describes how XMPP enables inter-module and inter-domain communications in IoT Cloud environments.

### 4.1. XMPP Overview

A valuable solution for the implementation of the communication system of a piece of IoT Cloud middleware, designed according to the MOM4C architectural model, is to adopt an instant message-oriented approach. In this regard XMPP, also called Jabber, is becoming more and more popular due to its flexibility to suit different scenarios in which a high-level of re-activeness is strongly required. Although it was born for human interaction via chat room (it is currently used in WhatsApp) it can be used to develop the communication of whatever distributed system well fitting the requirements of IoT. XMPP is an XML-based protocol used for near/real-time, extensible instant messaging and presence information. XMPP remains the core protocol of the Jabber Instant Messaging and Presence technology. The “Jabber” technology leverages open standards to provide a highly scalable architecture that supports the aggregation of presence information across different devices, users and applications. Like email, anyone who has a domain name and an Internet connection can run the Jabber server and chat with other users. The Internet Engineering Task Force (IETF) has formalized XMPP as an approved instant messaging and presence technology, and the specifications have been published as RFC 3920 and RFC 3921.

XMPP offers many advantages for the design of the communication system of complex distributed system. In the panorama of IoT Clouds, XMPP represents a flexible solution allowing to built custom functionalities. In this regard, common extensions are managed by the XMPP Software Foundation. XMPP provides a technology for asynchronous end-to-end exchange of structured data. Considering a distributed system, the protocol allows to build one or more overlay networks having global addressing (JIDs), network availability (presence), concurrent information transactions, distributed federated networks, structured data with XML payload. The architecture is similar to the email network, but it introduces several added value features to facilitate near-real-time communications. The end-to-end communication in XMPP is logically peer-to-peer. If it is assumed that each server can manage a domain, a server-to-server connection can enable inter-domain federation. Through XMPP, data are sent over persistent XML steams. XMPP clients (i.e., human or software modules) are connected over a Multi User Chat (MUC) room which represents a sort of broadcast domain. Summarizing, XMPP presents several advantages compared to web services including:

- Support to End-to-end communication;
- Real-time capabilities such as heartbeat, alarms, and any kind of asynchronous communication;
- Efficient distribution of data with public/subscribe and end-to-end approaches;
- Advanced service discovery;
- Federation. Most firewalls allow users to fetch and post messages without hindrance. Thus, if the Transmission Control Protocol (TCP) port used by XMPP is blocked, a server can listen on the normal HTTP port and the traffic should pass without problems.

### 4.2. Management of Inter-Module Communications

When two different IoT device have to interact each other, the inter-module communication has to be exploited. The typical use cases refer to:

- Communication between CM and TE for the exchanging of information related to the cluster status and for enforcing specific commands;
- Communication between the administrators and CM using the ad-hoc client interface.

**Figure 3:**
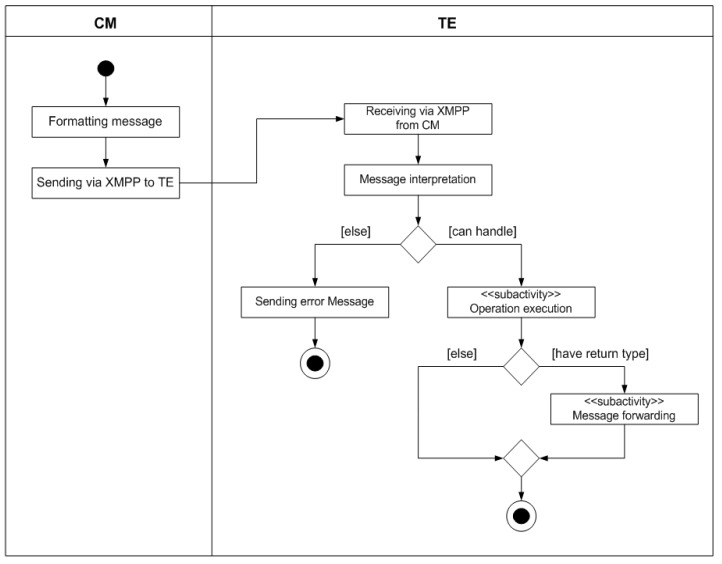
Activity diagram of the external communication.

In order to implement the inter-module communication mechanism, an XMPP server must exist within the MOM4C domain and all its entities must be connected to the same XMPP room. The XMPP server can be directly installed in IoT devices. When a message has to be transmitted from the CM to an TE, as represented in Figure 3, it is formatted and then sent using XMPP. Once received, the message is checked from the TE, for verifying if the requested operation can be performed.

**Figure 4:**
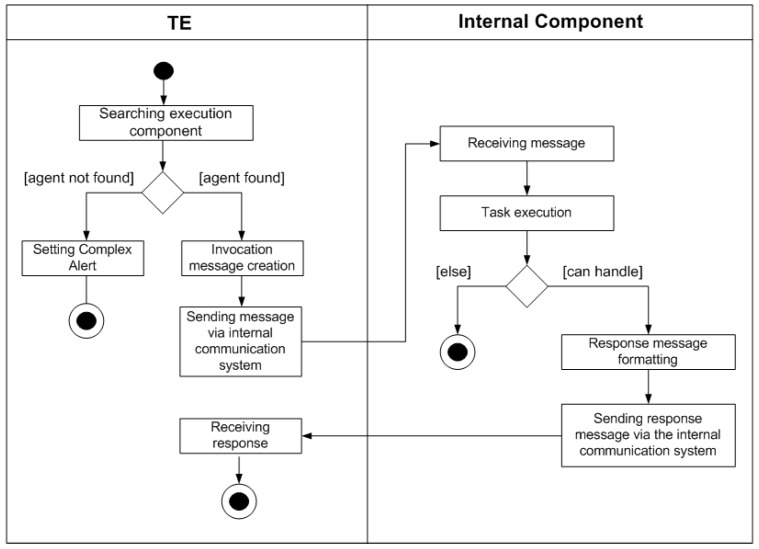
Activity diagram of the sub-activity executing operation.

As depicted in Figure 3, two different situations could occur: if a request can be handled, it is performed sending eventually an answer to the CM (if a return value is expected), otherwise an error message will be sent specifying an error code. The “Execution Operation” is a sub-activity whose description is pointed out in Figure 4. When the sub-activity is performed, if any return value is expected the procedure terminates, else this value has to be forwarded to the CM in the same way it has been done previously with the request. The sequence of steps involved in the sub-activity is shown in Figure 4. If the operation that has to be executed involves a component different from the TE, the already described intra-module communication has to be employed. Once the selected component receives the message using this mechanism, if no problem occurs, the associated activity will be performed, else an error will be generated. If the operation is executed correctly and a return value has to be generated, the component will be responsible to generate the response message which will be forwarded to the TE, and thus, to the CM.

### 4.3. Management of Inter-Domain Communications Enabling IoT Federation

The MOM4C architectural model enables federation between different IoT Cloud providers. Federation allows IoT Cloud providers to “lend” and/or “borrow” sensing and actuating resources to/from other IoT Cloud providers [1]. This means that a CM of an IoT Cloud administrative domain is able to control one or more TEs belonging other IoT Cloud administrative domains. For example, if an IoT Cloud of domain B runs out the sensing and/or actuating resources of its own TEs, it can establish a federation with an IoT Cloud of domain A, in order to allow the CM of domain B to use one or more TEs of the IoT Cloud domain A. This enables the CM of an IoT domain B to allocate sensing and/or actuating services both in its own TEs and in the TEs borrowed from domain A. In this way, on one hand the IoT Cloud of domain B can continue to allocate services for its clients (e.g., IT companies, organization, desktop end-users, etc), whereas on the other hand the IoT Cloud of domain A earns money from IoT Cloud of domain B for TEs renting.

**Figure 5:**
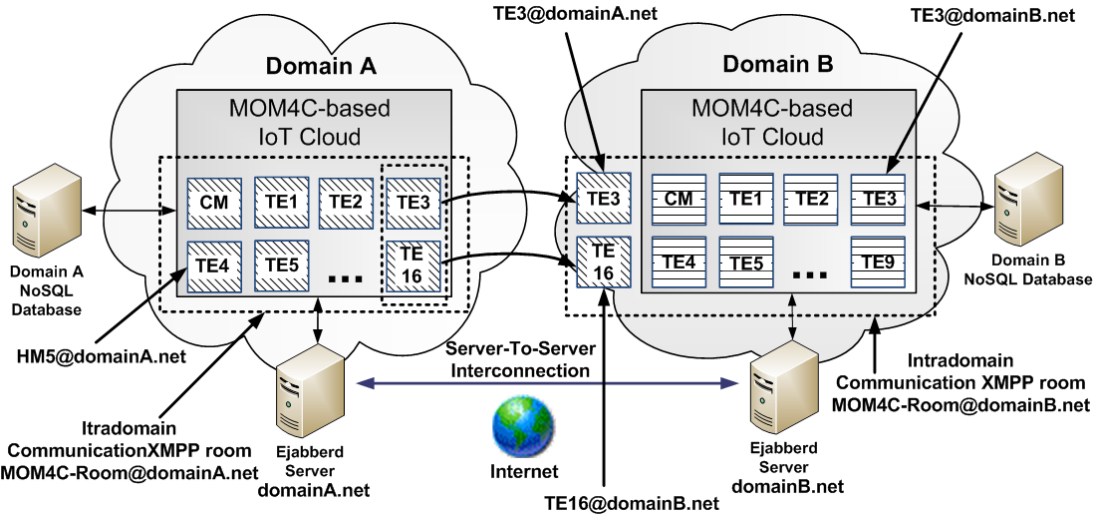
Example of federation between two MOM4C-based IoT Clouds.

In order to avoid to use a single server, eJabberd [32], i.e., one of the major XMPP server software solution, allows to organize the whole XMPP system in a distributed fashion by means a set of hierarchical servers. Since each IoT Cloud typically runs its own XMPP server on its own domain, an inter-domain communication is required among the two XMPP servers in order to establish a federated communication. Usually, every user on the XMPP network has an unique Jabber ID (JID) that is structured similarly to an e-mail address with an user name and a domain name for the server where that user is placed, separated by an at sign (@).For example, considering the MOM4C scenario, a CM could be identified by the CM1@domainB.net JID, whereas a TE could be identified by the TE2@domainA.net JID: CM1 and TE2 respectively represent the host names of the CM and the TE, instead domainB.net and domainA.net respectively represent the domains of the IoT Cloud that “borrows” its TEs and of the IoT Cloud which “lends” TEs. Let us suppose that CM1@domainB.net wants to communicate with TE2@domainA.net, CM1 and TE2, each respectively, have accounts on domainB.net and domain A XMPP servers. Figure 5 depicts an example of MOM4C inter-domain communication between two administrative domains for the renting of two TEs from an IoT Cloud domain A to an IoT Cloud domain B.

The two domains are identified by domainA.net and domainB.net identifiers. In a scenario without federation, they respectively manage different XMPP MUC for intra-domain communication (that are, mom4cRoom@domainA.net and mom4cRoom@domainB.net) on which a single CM, responsible for the administration of the domain, communicates with several TEs, typically placed within the physical cluster of the IoT Cloud domain. Considering a scenario of federation between the two different IoT administrative domains, if the CM the domainB.net domain needs external sensing and/or actuating resources, after an a priori agreement, it can invite within its mom4cRoom@domainB.net room one or more TEs of the domainA.net domain. For example, as depicted in Figure 5, the IoT Cloud of domainB.net rents from the IoT Cloud of domainA.net, TE6 and TE16. Thus, the two borrowed TEs will be physically placed in domainA.net, but they will be logically included in domainB.net. As previously stated, in order to accomplish such a task a trust relationship between domainA.net and domainB.net XMPP servers has to be established to enable a server-to-server communication allowing TEs of domain A to join the external XMPP MUC of domain B.

## 5. Security Issues in XMPP-Based Communication Systems for Federated IoT Environments

In our opinion, a IoT Cloud environment must follows the Cloud Security Alliance (CSA)’s guidance directives as summarized below:

1. In the Governance and Enterprise Risk Management, there is the need to *“divulge policies, procedures and processes comprising the IoT Cloud providers’ Information Security Management System (ISMS)”*, knowing who makes what.
2. Whereas in the Information Management and Data Security, it is necessary to *“assure that IoT Cloud provider personnel controls are in place to provide a logical segregation of duties.”*
3. In the Traditional Security, Business Continuity and Disaster Recovery, *“Customers should perform onsite inspections of IoT Cloud provider facilities whenever possible.”*
4. Data Center Operation, *“IoT Cloud providers must all be able to demonstrate comprehensive compartmentalization of systems, networks, management, provisioning and personnel.”*
5. In the Incident Response, Notification and Remediation, *“IoT Cloud providers should construct a registry of application owners by application interface (Uniform Resource Locator (URL), Service Oriented Architecture (SOA) service, etc.)”*.
6. Encryption and Key Management, where *“segregate the key management from the ioT Cloud provider hosting the data, creating a chain of separation”*.

Considering as ejabberd as reference XMPP server implementation, even though XMPP supports both SASL and TLS mechanisms for the authentication and encryption of the communication channels between different XMPP ejabberd servers, it presents some security limitations due to the decentralized nature of the protocol that demands the accomplishment of specific security mechanisms to the different implementations. On the other hand, the flexible and extensible nature of the protocol allows to integrate basic security mechanisms, improving the level of the security in the communications. In particular, considering federation-enabled IoT Clouds, XMPP does not allow to natively develop the following security mechanisms:

- **Data Confidentiality, Integrity, and Non Repudiation for Message Exchange**. As previously discussed, the different software modules can communicate over one or more MUCs that allow to isolate the communication of the involved software modules also providing a way to control which module can join a chat-room by means of a username/password authentication. This level of security is particularly weak considering emerging IoT Cloud architectures. Considering software modules A and B of an IoT Cloud system (i) modules A and B have to perform a mutual authentication before communicating through X.509 certificates in order to avoid identity-thief attacks; (ii) message exchanged between software modules A and B has to be confidential and not corrupted in order to avoid man-in-the-middle attacks; (iii) if software module A sends a message to B, module A cannot deny of having done it.
- **SSO Authentication for IoT Cloud federation**. Federation between IoT Cloud providers implies the establishment of a secure inter-domain communication between the XMPP servers of the involved IoT environments. This raises several issues regarding the management of authentication between the XMPP servers of different IoT Cloud domains. In fact, considering a scalable scenario including *n* IoT Clouds in order to perform an inter-domain federation the XMPP server of each IoT Cloud should perform n−1 authentication on the other n−1, hence managing n−1 different credentials for accessing the federated IoT Clouds. Considering the whole IoT Cloud federation ecosystem it is required to manage n(n−1) different credentials. The Identity Provider/Service Provider (IdP/SP) scheme allows to address such a problem introducing a trusted third-party, i.e., the IdP, so that an IoT Cloud provider that wants to perform a federation with the other n−1 IoT Clouds has to perform the authentication once, gaining the access on the other n−1 IoT Clouds which will be trusted with the IdP. Unfortunately, at the moment of the writing of this paper, the SASL/TLS on which the XMPP is based does not support any standard form of SSO authentication for server-to-server federation.

In the following, the previously introduced security limitations are discussed in detail.

### 5.1. Concerns Regarding Data Confidentiality, Integrity, and Non Repudiation

Let us consider an IoT Cloud provider including several distributed software modules or components deployed on IoT devices and whose inter-module communication takes place by means of an instant messaging protocol, such as XMPP. The question is: which are the security requirements of the involved communication system? Definitely it should ensure: *data confidentiality*, *data integrity*, and *data non-repudiation of the sender/receiver module*. Let us assume that in order to achieve a totally secure communication system each message has to be signed and encrypted by each software module. Considering the aforementioned security requirements, XMPP has to be properly extended. In our opinion, considering a Public Key Infrastructure (PKI), the XMMP-based communication of an IoT Cloud system should support the following functionalities:

- **Digital identity management**. Each IoT device, acting as CM and/or TE node, during the in-band registration (i.e., an automatic enrolment of the IoT device on the XMPP server) with the XMPP server requires a digital certificate to a trusted Certification Authority (CA) through the Simple Certificate Enrollment Protocol (SCEP).
- **Signed message exchange**. Each IoT device, acting as CM and/or TE node, should be able to sign a message sent to another one.
- **Encrypted message exchange**. Each IoT device, acting as CM and/or TE node, should be able to perform a total or partial encryption of the message body.
- **Private chat rooms**. The communication system should allow the management of private MUCs with restricted access to authorized software modules.
- **Encrypted chat rooms**. The communication system should allow the management of private and encrypted MUCs. The key exchange between communicating modules should take place according to a PKI schema. The component that play the role of “moderator” instantiate a new MUC associating a session key. When a new component wants to join the communication, the “moderator” component sends the session key encrypted with the public key of the new component itself.

### 5.2. Concerns about XMPP Server-to-Server IoT Federation

Assuming that the communication in each MOM4C-based IoT Cloud is achieved through XMPP messages by means of an ejabberd server, the federation establishment between two or more IoT Clouds implies a secure server-to-server inter-domain communication between their respective ejabberd servers. Moreover, according to the XMPP terminology, the term “federation” is commonly used to describe the communication between two servers. According to the MOM4C architectural model, each IoT Cloud belongs to a domain managed by an ejabberd server. Thus, according to MOM4C, the way to federate two IoT Clouds is to establish a secure communication among the ejabberd servers of the involved administrative domains. Access control policies on software modules and IoTaaS are out of the scope of this paper.

Cloud federation raises many issues especially in the field of security and privacy. Single Sign On (SSO) authentication is fundamental for the achievement of security features in a scalable scenario such as in a federated IoT environment. However, SASL (i.e., a framework for authentication and data security in Internet protocols) supported by XMPP does not allow any SSO authentication mechanism. In this regard, even though the public-subscribe technology is re-emerging for enabling real-time communication in IoT environments, XMPP is somewhat dated from the point of view of security. In order to enable federation between the ejabberd servers of different IoT Cloud providers, it is required to carry out a strong security to ensure both authentication and confidentiality by means of encryption mechanisms. In fact, according to the IETF 6120, compliant implementations of servers should support a Dialback [33] or a SASL EXTERNAL protocol for authentication and the TLS protocol for encryption. The basic idea behind Server Dialback is that a receiving XMPP server does not accept traffic from a sending XMPP server until it has (i) “called back” the authoritative server for the domain asserted by the sending server and (ii) verified that the sending server is truly authorized to generate XMPP traffic for that domain. The basic flow of events in Server Dialback consists of the following four steps:

1. The originating server generates a Dialback key and sends that value over its XML stream to the receiving server. (If the originating server does not have yet an XML stream with the receiving server, it will first need to perform a Domain Name System (DNS) lookup on the target domain and after that it has discovered the receiving server, it opens a TCP connection using the discovered IP address and port, and finally establishes an XML stream with the receiving server.)
2. Instead of immediately establish a MUC using the connection established by the originating server, the receiving server sends the same Dialback key over its XML stream with an authoritative server for verification.
3. The authoritative server informs the receiving server whether the key is valid or invalid.
4. The receiving server informs the originating server whether its identity has been verified or not.

As previously mentioned, SASL is a framework for providing authentication and data security services in connection-oriented protocols via replaceable mechanisms. It provides a structured interface between protocols and mechanisms. The resulting framework allows new protocols to reuse existing mechanisms and allows old protocols to make use of new mechanisms. SASL is used in various application protocols (e.g., XMPP, Internet Message Access Protocol (IMAP), Lightweight Directory Access Protocol (LDAP), Simple Mail Transfer Protocol (SMTP), Post Office Protocol (POP), etc.) and support many mechanisms including:

- **PLAIN**, a simple clear text password mechanism. PLAIN obsoleted the LOGIN mechanism.
- **SKEY**, an S/KEY mechanism.
- **CRAM-MD5**, a Challenge-Response Authentication Mechanism based on the keyed-Hash message Authentication Code (HMAC) MD5 algorithm, a simple challenge-response scheme based on TEAC-MD5.
- **DIGEST-MD5**, an HTTP Digest compatible challenge-response scheme based upon MD5 that offers a data security layer.
- **GSSAPI**, a Kerberos V5 authentication via the GSSAPI that offers a data-security layer.
- **GateKeeper**, a challenge-response mechanism developed by Microsoft for MSN Chat.

At the time of writing of the IETF 6120, most server implementations use the Dialback protocol to provide weak identity verification instead of using SASL to provide strong authentication, especially in cases where SASL negotiation would not result in strong authentication anyway (e.g., because the TLS negotiation was not mandated by the peer server or because the Public-Key Infrastructure X.509 (PKIX) certificate presented by the peer server during TLS negotiation is self-signed and has not been previously accepted). The solution is to offer a stronger level of security through SASL and TLS.

## 6. Securing the XMPP-Based Communication System for Federation-Enabled IoT Clouds

In this Section, considering an IoT Cloud environment according to the MOM4C architectural model, we discuss how can be possible to enforce Message Signing/Encryption for inter-module communication and SASL/SAML SSO authentication for inter-domain communication.

### 6.1. Message Signing/Encryption for Inter-Module Communication

Custom functionality can be built on top of XMPP by adding common extensions managed by the XMPP Software Foundation. Regarding security, even if the XMPP specification support both SASL and TLS technologies for the authentication and encryption of communication channels, it presents some limitations due to the decentralized nature of the protocol that demands the accomplishment of specific security mechanisms to the different implementations. On the other hand, the flexible and extensible nature of the protocol allows to integrate basic security mechanisms, improving the level of security in the communications.

As previously discussed, in order to guarantee data confidenziality, integrity, and non repudiation in an XMPP-based communicatin system of a federation-enabled IoT Cloud, specific security extensions are required. The *XEP 0027* [34] specification describes the use of Jabber with the Open Pretty Good Privacy (OpenPGP-RFC 4880-[35]). OpenPGP is an interoperable specification that provides cryptographic privacy and authentication for data communications. As highlighted by the *Internet draft*, XEP 0027 does not represent a standard, although it could be in the future, but it describes a possible solution for authentication and data encryption in end-to-end XMPP communications.

XEP 0027 allows the addition of specific XML tags in the XMPP message, each one defined by a specific *namespace*: for example *“jabber:x:signed”* and *“jabber:x:encrypted”* as shown respectively in Listing 1 and Listing 2. Such tags, indicate to the system how to process the information contained within them. As suggested in the specification, it is possible to apply the digital sign of a sender software module to a message, for example by using its private key. The specification, also allows the system to sign the presence message in a MUC coming from a specific software module. In this way, it is possible to sign the status of the sender module. In the following, an example of secure XMPP message sent from the *TE1* to *TE2* is discussed. As shown in Listing 1, the status of *TE1* is signed with her private key, so that *TE2* can verify by means of the *TE1*’s public key that it is really online. In the same way, it is possible to encrypt the content of the tag body using the public key of the receiver in order to achieve confidentiality. Listing 2 shows a message sent from *TE1* to *TE2* whose content has been encrypted with the public key of *TE2*. The specification does not define the exchange of public keys that is demanded to OpenPGP. Even though the chat messaging is something that purely seem regarding the human interaction, the same approach can by applied to Cloud computing systems in which different distributed software components need to interact each others in both real time and in secure way.

Listing 1 **Example of XEP 0027 XMPP message signing.**

<presence from=‘TE1@domainA.com’

     to=‘TE2@domainB.com’>

 <status>Online</status>

 <x xmlns=‘jabber:x:signed’>

   iQA/AwUBOjU5dnol3d88qZ77EQI2JAC

   fRngLJ045brNnaCX78ykKNUZaTIoAoP

   HI2uJxPMGR73EBIvEpcv0LRSy+=45f8

 </x>

</presence>

Listing 2 **Example of XEP 0027 XMPP message encryption.**

<message to=‘TE1@example1.com’

      from=‘TE2@example2.com’>

 <body>This message is encrypted.</body>

    <x xmlns=‘jabber:x:encrypted’>

      qANQR1DBwU4DX7jmYZnncmUQB/9KuKBdd

      zQH+tZ1ZywKK0yHKnq57kWq+RFtQdCJWp

      dWpR0uQsuJe7+vh3NWn59/gTc5MDlX8dS

      9p0ovStmNcyLhxVgmqS8ZKhsblVeuIpQ0

      JgavABqibJolc3BKrVtVV1igKiX/N7Pi8

      RtY1K18toaMDhdEfhBRzO/XB0+P

    </x>

 </body>

</message>

In order to secure the inter-module communication, it is needed to integrate public key infrastructure (PKI), Simple Certificate Enrollment Protocol (SCEP), Certification Authority (CA), and The Lightweight Directory Access Protocol (LDAP) mechanisms. In order to achieve a secure inter-module communication, it is mandatory to have a digital identity for each element. For this reason, during the initialization of each entity (e.g., CM, or TE), it is needed to setup the corresponding digital identity. Each entity obtains through the SCEP a private/public key pair from the CA. After that, it creates a KeyStore local object, in which each requesting entity can find, protected by password, its private key and the digital certificate in PKCS# format. After that the certificate is published on the LDAP server acting as “publisher” of the digital certificates associated to various software modules. When a module has obtained its own digital identity and it can access the LDAP server storing the public keys of the other entities, it is able to establish a secure inter-module communication with other software modules. Thus, both CM and TE are able to sign a message with its private key and to encrypt target message contents. In the first case the receiver module (for example the TE2 node) will be able to verify the digital sign of the sender (for example the TE1 node) by means of the corresponding public key read from the LDAP server. In the second case, a module (for example the TE1 node) will be able to use the PKI infrastructure in order encrypt the body of a message sent to another module (for example the TE2 node). In particular, the sending module will negotiate a shared key in order to encrypt data according to a symmetric cryptography scheme. In fact, it is remarked that the symmetric encryption is more performing than an asymmetric one from a computational point of view.

**Figure 6:**
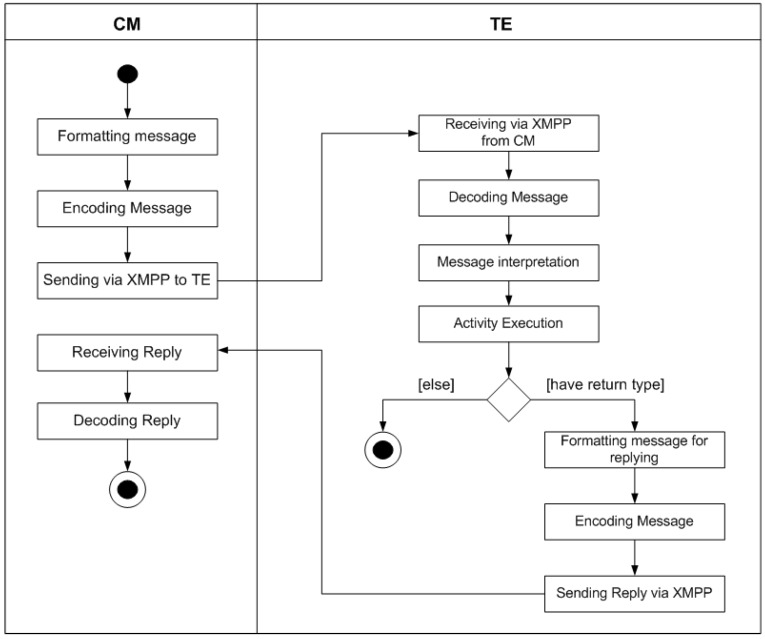
Secure Inter-module communication.

Figure 6 shows the activity diagrams of the secure inter-module communication with authentication/encryption.

**Figure 7:**
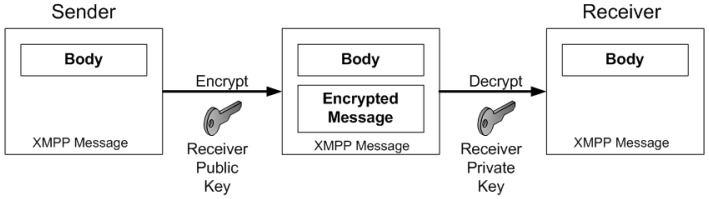
XMPP message encryption in MOM4C.

In order to guarantee data confidentiality, integrity, and non-repudiation in the XMPP-based communication system of a federation-enabled IoT Cloud, four basic extensions are required in the XMPP messages:

- **Signed**. It allows to attach to the message body a digest signed with the private key of the sender component. The signed extension is identified by the XML name space jabber:x:signed (*<x xmlns=‘jabber:x:signed’>*) known by all the components. When the message arrives to the receiver software module, it detects the signed extension and it queries the LDAP publisher server if an X509 certificate exists for the sender. If it exists, the receiver validates the sign and verifies the message digest according to a shared algorithm.
- **Encrypted**. It allows to attach to the message body a content encrypted with the public key of the receiver module. When a component wants to send an encrypted message, it requests to the LDAP publisher server the X509 certificate of the receiver component. Thus using the public key of the receiver, the sender module encrypts the message and it includes the encryption extension identified by the “jabber:x:encrypted” name space (*<x xmlns=‘jabber:x:encrypted’>*). When the message arrives to destination, the receiver component decrypts it with its private key. This process is summarized in Figure 7.
- **Session Key**. It allows to attach to the message a session key. It is used to support a hybrid encryption scheme: the unique shared key or the session key is used to encrypt/decrypt the messages sent by sender and receiver modules according to a symmetric encryption scheme (already used in the SSL/TLS protocol), but the session key is exchanged between the two parties according to a public key or asymmetric schema. The advantages of such a hybrid cryptographic scheme is twofold: session key secrecy and faster processing during the encryption/decryption of the message body.
- **Timestamp**. It allows to attach to the message a signed timestamp in order to enable an investigative support.

### 6.2. SASL/SAML SSO Authentication for Inter-Domain Communications

In a scalable scenario of federation each IoT Cloud can require to frequently establish/break partnerships with other IoT Clouds. This implies that each IoT Cloud should manage a huge number of credentials in order to authenticate itself in other IoT Clouds. In a federated environment, this means that the XMPP server of the IoT Cloud requiring federation has to be authenticated by the XMPP server of the Cloud accepting the federation request. If thousand of IoT Clouds are considered, each IoT Cloud should manage one credential for accessing to each specific federated IoT Cloud. This problem is commonly known as SSO authentication, i.e., considering an inter-domain environment, it allows an IoT Cloud to perform the authentication once, gaining the access to the resources supplied by other federated IoT Cloud provider, each one belonging to a specific domain. A previously mentioned, a model for addressing the SSO problem is IdP/SP. Typically, a client who wants to access the resources provided by a SP, perform the authentication once on the IdP (asserting party), which asserts to the SP (relaying party) the validity of the authentication of the client. Considering many SPs relaying on the IdP if the client wants to access another SP, as this latter will be trusted with the IdP, no further authentication will be required. This model is widely adopted on the web with the term “Web Browser SSO”, in which the client is commonly an user who performs an authentication filling in his/her user name and password by means of an HTML form. Nowadays, the major standard defining the IdP/SP model is the Security Assertion Markup Language (SAML) [36], developed by the Advancing Open Standards for the Information Society (OASIS).

The scenario of IoT Cloud federation is quite similar. In this case, the client who wants to perform the authentication is the eJabberd server of the IoT Cloud requiring federation, instead the role of the SP is played by the eJabberd server of the IoT Cloud accepting the federation request. As the eJabberd server supports authentication through SASL a concern raises: the RFC 4422 does not support any security mechanism implementing the IdP/SP model. Therefore, in order to achieve such a scenario, the Internet-Draft entitled “A SASL Mechanism for SAML”, defined by CISCO TF-Mobility Vienna, was considered, describing the applicability and integration between the two protocols for non-HTTP use cases. According to such a draft, the authentication should occur as follows:

1. The server may advertise the SAML20 capability;
2. The client initiates a SASL authentication with SAML20;
3. The server sends the client one of two responses:
  (a) a redirect to an IdP discovery service; or
  (b) a redirect to the IdP with a complete authentication request;
4. In both case, the client must send an empty response;
5. The SASL client hands the redirect to either a browser or an appropriate handler (either external or internal to the client), and the SAML authentication proceeds externally and opaquely from the SASL process;
6. The SASL Server indicates success or failure, along with an optional list of attributes.

In this way, thanks to a combination between SASL and SAML, each IoT Cloud provider is able to perform the authentication once gaining the access to all the other IoT Clouds relying on the same IdP, thence, lending and/or borrowing TEs according to a priori agreements.

Two different levels of XMPP server-to-server federation exist:

- Permissive Federation, a server accepts a connection from any other peer on the network, even without verifying the identity of the peer based on DNS lookups.
- Verified Federation, a server accepts a connection from a peer only after that its identity has been weakly verified via Server Dialback, based on information obtained via the DNS and verification of the keys exchanged in-band over XMPP.
- Encrypted Federation, a server accepts a connection from a peer only if the peer supports TLS and the client authenticates itself using a SASL mechanisms.

On one hand, Permissive and Verified Federation are the simplest federation approaches. However, they lack of some security aspects since they are not based on any password exchange procedure in order to implement domain filtering (in the second case) and a list of allowed domains has to be a priori defined. On the other hand, the Encrypted Federation level relies on a more secure way to perform the authentication, based on challenge-response authentication protocols relaying on passphrase. This standard authentication mechanisms are enough when an IoT Cloud needs to enable the communication with a limited number of other IoT Cloud providers, but in a scenario where several XMPP servers might exist, it could be a difficult task to be managed. A possible solution consists in integrating SASL with a SSO authentication mechanism based on SAML 2.0.

**Figure 8:**
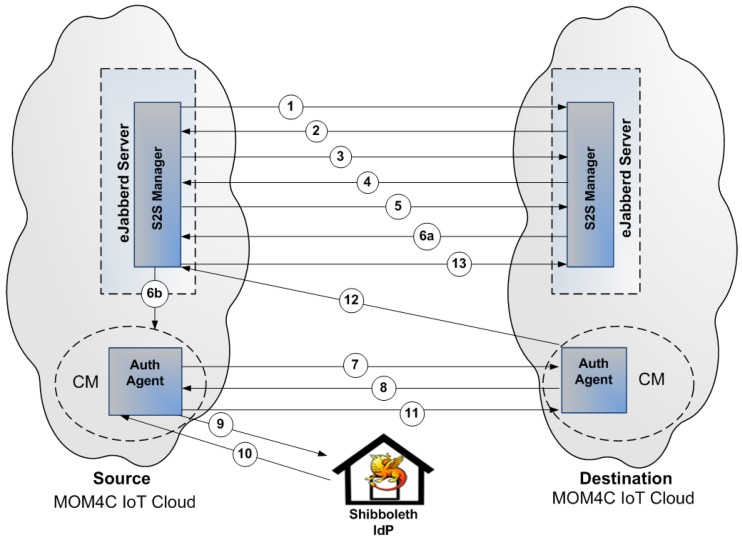
Step performed by two XMPP servers aiming to build an IoT Cloud federation: the authentication process is executed using SAML 2.0 as external SASL mechanism.

Let us consider two IoT Cloud providers each one relying on its own eJabberd XMPP server enabling the communications within the domain. Generally the server-to-server federation is accomplished by an eJabberd module that manages incoming and outcoming connections from/to external eJabberd servers. According to the XMPP core specification, this module is able to establish server federation according to the three different federation levels pointed out above. In order to enable eJabberd servers to perform SSO authentication the Encrypted Federation case has to be considered, extending the eJabberd module that performs SASL in order to add in the list of the supported security mechanisms also SAML 2.0. For simplicity, such a module was named Server-to-Server (S2S) Manager. A possible way for the achievement of this goal is the implementation of the Internet-Draft entitled “A SASL Mechanism for SAML” defined by CISCO TF-Mobility Vienna relying on an external software module based on Shibboleth, that was named Authentication Agent (AA). Considering the MOM4C architectural model, the AA is controlled by the CM node and acts as user when it is contacted from the S2S manger module of its domain for starting the IoT federation. On the contrary, it acts as relying party when it is contacted from another IoT Cloud provider. In the following, the sequence of steps performed by two eJabberd servers to establish a federated connection is described. As Figure 8 depicts, the involved actors in the process are the S2S Managers of both eJabberd servers, the two AAs respectively acting as user in the Source MOM4C IoT Cloud provider and relying party in the Destination MOM4C IoT Cloud provider. In this example, the Identity Provider is represented by the Shibboleth server.

- Step 1: S2S Manager of Source eJabberd Server initiates stream to the S2S Manager of the Destination eJabberd Server.
- Step 2: S2S Manager of the Destination eJabberd Server responds with a stream tag sent to the S2S Manager of the Source eJabber Server.
- Step 3: S2S Manager of the Destination eJabber Server informs the S2S Manager of the Source eJabberd Server of available authentication mechanisms.
- Step 4: S2S Manager of the Source eJabberd Server selects SAML as an authentication mechanism.
- Step 5: S2S Manager of Destination eJabberd Server sends a BASE64 encoded challenge to the S2S Manager of the Source eJabberd Server in the form of an HTTP Redirect to the Destination AA (acting as relying party).
- Step 6: (a) S2S Manager of Source eJabberd Server sends a BASE64 encoded empty response to the challenge; and (b) forward to the Source AA the URL of the relying party.
- Step 7: The Source AA (acting as user) engages the SAML authentication flow (external to SASL) contacting the Destination AA (acting as relying party).
- Step 8: Destination AA redirect Source AA to the IdP.
- Step 9: Source AA contacts IdP and performs Authentication.
- Step 10: IdP responds with Authentication Assertion.
- Step 11: Source AA contacts Destination AA for gaining access to the resource.
- Step 12: Destination AA contacts the S2S Manager of the Destination eJabberd Server informing it about the authentication result.
- Step 13: if the authentication is successful the S2S Manager of the Source eJabberd Server initiates a new stream to the S2S Manager of Destination eJabberd Server.

The advantage of performing the authentication among servers in such a way is mainly the higher security level achieved than traditional Dialback/SASL mechanisms and in the possibility of exploiting the SSO authentication. Looking at Figure 8, after that the federation has been achieved, if the same Source IoT Cloud aims to perform server-to-server federation with a new XMPP server that relies on the same IdP as trusted third-party, such a process would be straightforward. Since the Source Server already has an established a security context with the IdP, once the SASL process starts and the SAML mechanism is selected, no further authentication will be required.

## 7. Experimental Results

In order to evaluate the impact of the advanced XMPP security mechanisms, a testbed was arranged in order to analyse the behaviour of the system in terms of both scalability and efficiency. Our testbed includes four Raspberry PI devices with following hardware configuration: system on-chip: Broadcom BCM2835, CPU: 700 MHz ARM11 ARM1176JZF-S core, memory: 512 MiB SDRAM, on-board storage: Class 10 micro 8GB SDHC card, on-board network: 10/100 wired Ethernet RJ45 connection, Operating System: Raspian.

Moreover, adapting the CLEVER software distribution [37], two administrative domains compliant with the MOM4C architectural model were implemented. In domain A, two devices respectively acting as client and CM were configured, whereas in domain B other two devices respectively acting as CM and TE were arranged. Furthermore, in each domain a dedicated eJabberd server to enable XMPP communications was configured. The testbed also included an LDAP server storing the public keys associated to devices, a Certification Authority and a Shibboleth IdP server. In order to establish a federated communication between the two domains, the eJabberd server of domain A was authenticated in the eJabber server of domain B through a SAML/SASL SSO authentication procedure. In this way, the TE of domain B was also included in domain A, by exploiting the concept of MUC. So as to simulate data traffic in our testbed, the domain A client device submitted to domain A CM a *listsensors* command for querying all sensors available on domain B TE. In order to simulate clients, in the client device, a specific thread for each user in the client device was instantiated. Each client thread submitted a listsensorsquery at a random time in the range of $\left[0;1\right]$ s, thus to simulate concurrent requests. This type of scenario causes a high overhead in the client device, when the number of users increases since threads independently work generating many messages in a short time interval. Such a scenario well describes a typical IoT service broker that works as collectors for client requests.

The system evaluation was performed according to the transmission time necessary to transfer a formatted message from a source to a destination device. Moreover, the information on the communication overhead was also provided. In particular, from 5 to 40 concurrent users’ requests were considered in order to understand when the security capabilities affect the performance of the Raspberry PI. More specifically, client to CM and CM to TE communications were analysed. Each experiment was repeated 50 times in order to consider mean values and achieving a confidence interval at least of 95%. The overhead due to security extensions in XMPP messages in terms of delay experienced in transferring data was assessed. Since the testbed uses GigaByte (GB) links, for simplicity, the contribution due to network latency was neglected. Considering a WAN scenario with a given network latency, these results can be used to evaluate any particular scenario.

**Figure 9:**
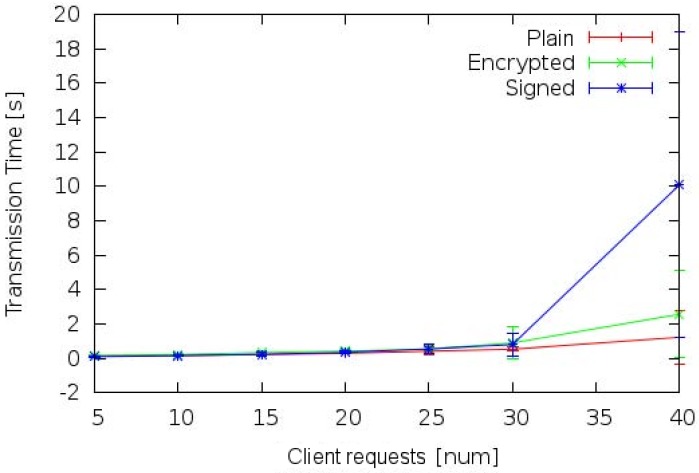
Transmission time between client and CM devices on domain A.

Figure 9 shows the mean transmission time of messages exchanged between client and CM devices. In particular, plain, signed and encrypted messages were considered. Different threads in the client devices worked in parallel sending their requests independently from each other. Increasing the number of clients, also the transmission time increases due to the higher number of messages generated in a short time interval and stored in transmission buffers. This effect is amplified when security mechanisms are considered. Data encryption implies the recovery of the public key of the destination in the LDAP server. Thus, transmission buffers hold both data messages and LDAP requests. Up to 30 concurrent users’ requests the signing and encryption overhead is negligible, but from 31 concurrent users’ requests the the situation changes due to a device overloading. Encrypted transmissions delays increase roughly 15 times according to the number of users (from 0,16 to 2,56 s), but an even worst behavior characterizes the transmission of signed messages, in which transmission time increases more than 100 times (from 0,10 to 10,09 s). Thus, the Raspberry Pi is not able to manage 40 concurrent signed messages with an acceptable time. This result is caused by the message size that grows with the sign digest. Applying SHA-1 to an arbitrary-length message *m* it produces a 20 bytes hash. Furthermore, the RSA algorithm includes an additional block of 128 bytes, with a total message digest length of 148 bytes. Since, in our tests, the message is very small (about 1-2 KBytes), the digital sign raises the data size of about 10%, causing a fast filling of the buffers. This result proves that up to 30 concurrent requests, security does not significantly affect the communication.

**Figure 10:**
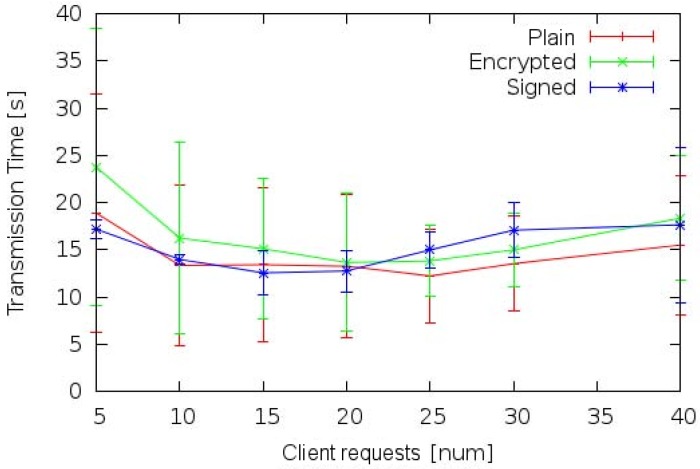
Trasmission time between domain A CM and domain B TE.

Figure 10 shows the mean transmission time of messages exchanged between domain A CM and domain B TE devices. It was experienced that message signing and encryption overheads are minimal even considering 35 and 40 concurrent users’ requests with little differences between observed response times. In addition, in a few cases (considering 5, 15 and 20 concurrent requests), it is observed that the communication times of plain text messages are even slowly greater than the transmission times of signed messages. For example, considering 15 concurrent requests the transmission time of plain text is greater than the transmission time of signed messages of roughly 100 milliseconds. This anomaly is probably due to the internal concurrent management tasks performed by the adopted piece of middleware during experiments. The description of message management performed by the middleware is out of the scope of this paper (further details are available in [37]).

Summarizing, the results derived from our prototype show that the proposed security mechanisms assuring confidentiality, authenticity and non-repudiation of data cause inevitable delays. However, such delays are still acceptable in absolute terms and determine a reasonable impact on all the management activities of a federated IoT Cloud provider.

## 8. Conclusions and Future Work

Currently, the major IoT Cloud solutions base their communication systems on HTTP-based web services that do not well suit the requirements of new emerging federated IoT Cloud architectures and services. From an analysis of literature, even if MQTT seams to be the best solution in terms of latency, it was highlighted that things are different considering both message management and security. In fact, MQTT is a client/server public/subscribe technology that does not support direct point-to-point and multicast messaging. As far as security, MQTT only supports SASL and TLS mechanisms respectively for authentication and for the encryption of the communication channel. On the contrary, XMPP seams to be more suitable for addressing the requirements of emerging IoT Cloud providers in terms of both message management and security. In fact, it is able to overcome the disadvantages of MQTT in terms of management because, apart from the public/subscribe communication, it also supports end-to-end and multicast communications. However, considering security and privacy, similarly to MQTT, XMPP supports only SASL and TLS capabilities, but it lacks of native advanced security features including: End-to-End and multicast message signing/encryption for inter-module communication and Cloud-to-Cloud authentication for inter-domain authentication.

In this paper, two solutions for carrying out such security mechanisms were presented. Regarding the inter-module communication, it was discussed how to extend XMPP for enabling signing/encryption mechanisms in message exchange according to the XEP 0027 specification, whereas, considering the inter-domain communication, it was proposed an approach based on SSO authentication for XMPP server-to-server federation according to the Internet-Draft entitled “A SASL Mechanism for SAML”.

Experiments conducted on a real testbed by extending the CLEVER middleware, prove that the overhead of message signing and encryption is negligible in terms of average transmission time.

In conclusion it is possible to state that XMPP is a valuable solution for the development of flexible and secure federation-enabled IoT Cloud systems in different critical context including e-health, e-business, etc.

We hope we succeed to alleviate the security gap for the of the XMPP in federation-enabled IoT Cloud architectures. In future works, we plan study secure self-identification mechanisms, that enable IoT devices joining a specific IoT Cloud provider to self-configure them by means of the secure deployment of container including specific software modules and utilities.
